# ENT3 utilizes a pH Sensing Mechanism for Transport

**DOI:** 10.1080/19336950.2017.1389581

**Published:** 2017-11-15

**Authors:** Anusha Singh, Rajgopal Govindarajan

**Affiliations:** a Division of Pharmaceutics and Pharmaceutical Chemistry, College of Pharmacy, The Ohio State University, Columbus, OH 43210, USA; b Translational Therapeutics, Ohio State University Comprehensive Cancer Center, The Ohio State University, Columbus, OH 43210, USA

**Keywords:** Transporter, nucleoside, lysosome, transport, SLC29, ENT3, pH, sensor, acidic, pH-dependent

Equilibrative nucleoside transporter 3 (ENT3), encoded by the SLC29A3 gene, is the major acidic pH dependent nucleoside transporter responsible for maintaining nucleoside homeostasis in lysosomal, and potentially, in mitochondrial compartments.^1,2^ Coupled with that characteristic, it is also amongst the most frequently mutated nucleoside transporters, and is responsible for numerous human genetic disorders including H syndrome, Insulin-dependent diabetes (PHID), Faisalabad histiocytosis (FHC), Sinus histiocytosis with massive lymphadenopathy (SHML), Rosai Dorfman Disease (RDD) and skeletal dystrophy.^3^ Despite, recent studies recognizing many of the above mentioned ENT3 disorders as lysosomal-storage or mitochondria-like disorders, the molecular basis of ENT3 transport, and thus, the molecular pathogenesis of ENT3 disorders are unknown.

In our recently published study,^4^ we employed site-directed mutagenesis, homology modeling and ^3^H-adenosine flux measurements in ENT mutant RNA-injected Xenopus oocytes to identify the molecular mechanisms behind pH-dependent ENT3 transport of endogenous nucleosides. As seen in earlier studies,^2,5^ we investigated the response of ENT3 to pH by deleting the N-terminal 36 amino acids, enabling ENT3 to localize on the cell surface, and noticed a sharp decline in the activity of ENT3 in pH ranges above 6.5. To analyze pH sensitivity of the different members of the ENT family we derived an r value, Transport Flux at pH 7.4/ Transport flux at pH 5.5, elucidating that the most pH dependent nucleoside transporter is ENT3. Paradoxically, while ENT3 primarily functioned in acidic conditions, the functioning of hENT1 and 2 injected oocytes was still robust even at acidic pH values, contrary to the fact they are expressed in the cell membrane and typically encounter a higher pH range.

Although it was discovered that ENT3 in kinetoplastoid protozoans is electrogenic, it was unknown whether human ENT3 utilized proton co-transport for permeant translocation. Our study discerned the role of ENT3 as a non-electrogenic transporter and supported that proton transport is not concurrent with adenosine transport. This led us to hypothesize that the interactions between protons and amino acids in ENT3, rather than electrogenicity, are responsible for acidic pH-activated transportability. Consequently, we tested the pH sensing capacity of histidine residues in ENT3 since they exhibit a pKa at the precise pH range that ENT3 functions. Substitution with non-ionizable amino acid Ala caused a loss of transportability of ^3^H-adenosine into oocytes at pH ranges 5.5 and 7.4. However, in order to ensure that this attenuation of transportability resulted from the inability of His to sense pH, and not due to other possible attributes such as loss of substrate recognition and improper membrane targeting, we carried out subsequent substitution studies examining the positively charged, negatively charged and neutral amino acids at these positions in ENT3 in both pH 5.5 and 7.4. Unexpectedly, our results suggested that histidines play a relatively minor role in pH sensing. Next, we conducted extensive screening analyses of three classes of amino acids (ionizable, aromatic and slightly basic rare group residues) shown to function as pH-sensing residues in mammalian and bacterial cells. We narrowed down to two primary acidic residues, Glu447 and Asp219, found on opposite sides of the membrane that utilize a pH-sensing mechanism by closing and opening the translocation pore of ENT3 in response to the transport microenvironment. Further, to confirm Asp219 and Glu447 as the key pH sensors, amino acid substitutions with various other amino acid residues were performed to find that every substitution, with some exceptions, that was not Cys at position 219 and Gln at position 447 (isosteric to Asp and Glu, respectively) resulted in pH independent activity. This revealed a novel role played by amino acid residues Glu447 and Asp219 in dictating —based on the pH of the microenvironment— the permissible and impermissible conformations of ENT3.

**Figure 1.**
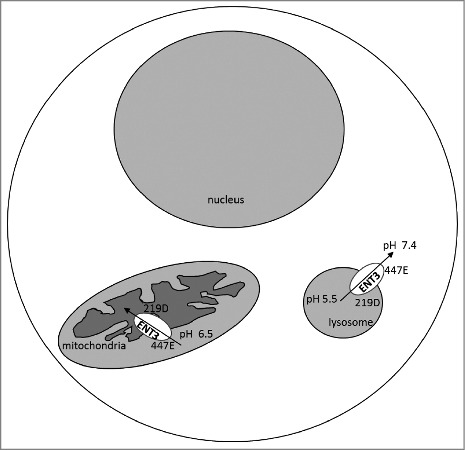
Schematic representation of pH-sensing involved in ENT3 organelle transport. In lysosomes, residue Asp219 faces the acidic-pH, while the Glu447 is exposed to the cytosolic pH. However, in mitochondria, the Glu447 is exposed to the relatively acidic mitochondrial intermembrane space. It is hypothesized that the transport-permissible conformational changes occur in ENT3 when the amino acid lying in direct contact with the acidic-pH microenvironment gets protonated. Putative directionality for nucleoside transport indicated.

Intriguingly, the topological locations of the Glu447 and Asp219 residues may provide an explanation about the ENT3 transport mechanism in lysosomes, and potentially in mitochondria. In lysosomes, Asp219 lies in direct contact with the acidic lysosomal interior and is predicted to play a more dominant role in pH-sensing activity than Glu447, which is exposed to a fairly steady pH cytosolic environment (Fig. 1). A reversal of roles of Glu447 and Asp219 is apparent when we study mitochondria, because unlike in lysosomes, Glu447 faces the mitochondrial intermembrane space, which is relatively acidic,^6^ while Asp219 is exposed to the slightly alkaline matrix. Thus, in the mitochondrial context, it is possible that Glu447 plays a predominant role that allows the import of nucleosides into the mitochondrial matrix (Fig. 1). The flipped roles of these residues in both the mitochondria and lysosomes hint at possibilities of crosstalk of transporter segments facing the inner membrane and the outer membrane, in turn influencing the directionality of nucleoside transport (mitochondrial influx of nucleosides versus lysosomal efflux of nucleosides). This may be of advantage from a cellular perspective to direct nucleosides from lysosomes to cytosol or cytosol to mitochondria to facilitate the fundamental function of salvage synthesis of nucleic acids. However, these remain as speculations at this stage and further studies are warranted to understand these processes.

Our findings on structural and functional characteristics of ENT3 through the identification of Glu447 and Asp219 as its primary pH sensors may also present pathophysiological implications. Coincidentally, around the time our work was published, another group investigated a case of H Syndrome that arose from a novel mutation (Glu447Lys) in the SLC29A3 gene.^7^ They hypothesized a link between the mutation and the symptoms experienced, ranging from various cardiovascular anomalies to skin-dominant features including hyperpigmentation and hypertrichosis. Our lab's research may hold an explanation for this molecular pathogenesis. Beyond mere substitution with the wrong amino acid residue, a transition from pH dependent transport to pH independent transport occurs in ENT3, possibly disrupting lysosomal homeostasis, and on a larger scale propagating molecular pathogenesis of H syndrome. In an earlier study,^8^ we established three possible consequences of ENT3 mutations: reduction or loss of nucleoside transport, loss of proper localization and a decrease in protein stability. However, linking our research on the pH sensing mechanisms of ENT3 with the aforementioned case study suggests the loss of pH sensitivity as a fourth possible consequence of ENT3 mutations. Furthermore, in addition to identifying pH sensitive residues, our study also highlighted several integral amino acid residues that remain key to ENT3 functioning, such as Glu444, one of the most commonly mutated residues associated with conditions like pigmented hypertrichosis with insulin-dependent diabetes mellitus syndrome or sinus histiocytosis with massive lymphadenopathy. Future studies investigating the precise roles of amino acid residues in ENT3 functionality are of considerable interest to improving our understanding about ENT3 disorders and promoting the creation of novel therapies and treatments to combat these disorders.
